# Unfolded protein response in cancer: the Physician's perspective

**DOI:** 10.1186/1756-8722-4-8

**Published:** 2011-02-23

**Authors:** Xuemei Li, Kezhong Zhang, Zihai Li

**Affiliations:** 1 Lea's Foundation Center for Hematologic Disorders and Neag Comprehensive Cancer Center, University of Connecticut School of Medicine, Farmington, CT 06030-1601, USA; 2 Center for Molecular Medicine and Genetics, Department of Microbiology and Immunology, Wayne State University, Detroit, MI 48201, USA; 3 Department of Microbiology & Immunology; Medical University of South Carolina, Charleston, SC 29425, USA

## Abstract

The unfolded protein response **(**UPR) is a cascade of intracellular stress signaling events in response to an accumulation of unfolded or misfolded proteins in the lumen of the endoplasmic reticulum (ER). Cancer cells are often exposed to hypoxia, nutrient starvation, oxidative stress and other metabolic dysregulation that cause ER stress and activation of the UPR. Depending on the duration and degree of ER stress, the UPR can provide either survival signals by activating adaptive and antiapoptotic pathways, or death signals by inducing cell death programs. Sustained induction or repression of UPR pharmacologically may thus have beneficial and therapeutic effects against cancer. In this review, we discuss the basic mechanisms of UPR and highlight the importance of UPR in cancer biology. We also update the UPR-targeted cancer therapeutics currently in clinical trials.

## 1. The unfolded protein response: mechanism

During tumorigenesis, the high proliferation rate of cancer cells requires increased activities of ER machinery in facilitating protein folding, assembly, and transport. Other pathologic stimuli can interrupt the protein folding process and subsequently cause accumulation of unfolded or misfolded proteins in the ER, a condition referred to as "ER stress" [1-5]. These pathologic stimuli include those that cause ER calcium depletion, altered glycosylation, nutrient deprivation, oxidative stress, DNA damage, or energy perturbation or fluctuations. In order to handle the accumulation of the unfolded or misfolded proteins, the ER evolves a group of signal transduction pathways, collectively termed the unfolded protein response (UPR), to alter transcriptional and translational programs to maintain ER homeostasis [6-8].

UPR has two primary functions: 1) to initially restore normal function of the cell by halting protein translation and activating the signaling pathways that lead to increased production of molecular chaperones involved in protein folding [9,10]; 2) to initiate apoptotic pathways to remove the stressed cells when the initial objectives are not achieved within a certain time lapse or the disruption is prolonged [11,12].

As a part of the UPR program, ER-associated Protein Degradation (ERAD) is responsible for the degradation of aberrant or misfolded proteins in the ER, providing an important protein folding "quality control" mechanism. During the process of ERAD, molecular chaperones and associated factors recognize and target substrates for retrotranslocation to the cytoplasm, where they are polyubiquitinated and degraded by the 26S proteasome [13]. ERAD is essential for maintaining ER homeostasis, and the disruption of ERAD is closely associated with ER stress-induced apoptosis [14].

Proteasomal degradation and autophagy have been identified as two main mechanisms in charge of protein clearance in stressed cells. Proteasomal degradation digests soluble ubiquitin-conjugated proteins. Autophagy involves cytoplasmic components engulfed within a double membrane vesicle (autophagosome). The maturation of these vesicles may fuse with lysosomes, which leads in turn to the degradation of the autophagosome components by the lysosomal degradative enzymes. Conditions that induce ER stress also lead to induction of autophagy [15]. Activation of the IRE1, phosphorylation of eIF2*α*, and ER Ca^2+ ^release can all regulate autophagy. Activation of autophagy after ER stress can be either cell-protective or cytotoxic. Persistent ER stress can switch the cytoprotective functions of UPR and autophagy into cell death programs. Some antitumoral agents (e.g., cannabinoids) activate ER stress and autophagy as the primary mechanism to promote cancer cell death [16-18].

### 1.1. The unfolded protein response pathways

**Figure 1:**
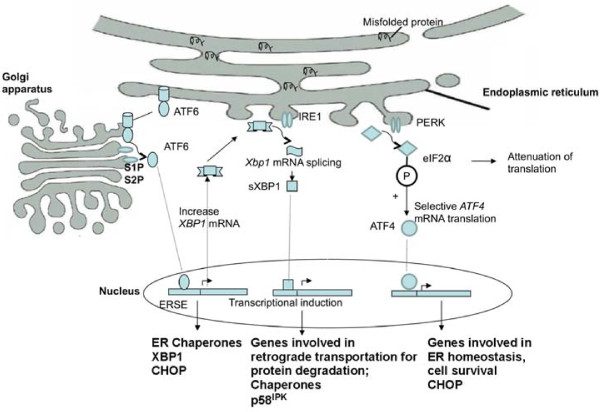
**Signal transduction events associated with ER stress and UPR**. Upon accumulation of unfolded or misfolded proteins in the ER three major ER stress sensors, PERK, ATF6 and IRE1, are activated following their dissociation from the ER chaperone GRP78. Activated PERK phosphorylates eukaryotic initiation factor 2α (eIF2α), which suppresses global mRNA translation but activates ATF4 translation. ATF4 translocates to the nucleus and induces the transcription of genes required to restore ER homeostasis. Activation of PERK also leads to the induction of CHOP (C/EBP homologous protein), which is involved in pro-apoptotic signaling. ATF6 is activated by proteolysis mediated by proteases S1P and S2P after its translocation from the ER to the Golgi apparatus. Active ATF6 translocates to the nucleus and regulates the expression of ER chaperones and X box-binding protein 1 (XBP1) to facilitate protein folding, secretion, and degradation in the ER. *Xbp1 *mRNA undergoes unconventional mRNA splicing carried out by IRE1. Spliced XBP1 protein (sXBP1) translocates to the nucleus and controls the transcription of chaperones, the co-chaperones and the PERK-inhibitor P58^IPK^, as well as genes involved in protein degradation.

On aggregation of unfolded proteins, GRP78 (known also as the immunoglobulin heavy chain binding protein, or BiP), one of the most abundant ER luminal chaperones, binds to unfolded proteins and dissociates from the three membrane-bound ER stress sensors. These stress sensors include pancreatic ER kinase (PKR)-like ER kinase (PERK), activating transcription factor 6 (ATF6), and inositol-requiring enzyme 1 (IRE1). The dissociation of GRP78 from these stress sensors allows their subsequent activation (Figure 1). It has been proposed that the activation of the ER stress sensors may occur sequentially, with PERK being the first, rapidly followed by ATF6, and IRE1 may be activated last [19].

Activated PERK blocks general protein synthesis by phosphorylating eukaryotic initiation factor 2α (eIF2α), which suppress mRNA translation. Reduced global translation also leads to reduction of key regulatory proteins that are subject to rapid turnover, facilitating activation of transcription factors such as NFκ-B during cellular stress [4]. However, selective translation of some proteins is activated, including ATF4, which occurs through an alternative translation pathway. ATF4, being a transcription factor, translocates to the nucleus and induces the transcription of genes required to restore ER homeostasis. Activation of PERK is initially protective and crucial for survival during mild stress. However, it leads to the induction of CHOP (C/EBP homologous protein), an important element of the switch from pro-adaptive to pro-apoptotic signaling [20-24].

PERK-mediated translational repression is transient and is followed by translational recovery and enhanced expression of genes that increase the capacity of the ER to process client proteins. P58^IPK ^induction during the ER-stress response represses PERK activity and plays a functional role in the expression of downstream markers of PERK activity in the later phase of the ER-stress response. P58^IPK^, GADD34 and TRB3, are reported to be involved in switching off the PERK mediated pathway. Blocking this protective pathway can be a central element of the switch from adaptation to apoptosis [19,25].

ATF6 is activated by regulated intramembrane proteolysis after its translocation from the ER to the Golgi apparatus [26]. Active ATF6 is also a transcription factor that regulates the expression of ER chaperones and X box-binding protein 1 (XBP1), another UPR-trans-activator. The target genes of ATF6 and XBP1 have been shown to be involved in protein folding, secretion, and degradation in the ER [27,28].

To achieve its active form, *Xbp1 *mRNA must undergo a non-conventional mRNA splicing, which is carried out by IRE1α. IRE1α protein is a type I transmembrane protein that contains both a Ser/Thr kinase domain and an endoribonuclease domain. The endoribonuclease domain processes an intron from the *Xbp1 *mRNA. Spliced XBP1 protein (XBP1s) translocates to the nucleus to activate the transcription of the genes encoding protein chaperones or folding enzymes involved in protein folding, secretion, or ERAD. Ablation of IRE1α in mice produces an embryonic lethal phenotype. It has been demonstrated that both processes of ATF6 activation and the IRE1α-mediated splicing of *XBP1 *mRNA are required for full induction of the UPR [29-31].

### 1.2. ER stress-induced apoptosis

The adaptive responses to the accumulation of unfolded or misfolded proteins in the ER provide initial protection from cell death. But persistent or excessive ER stress can trigger cell death, typically through apoptosis. Both mitochondria-dependent and -independent pathways have been proposed for ER stress-induced apoptosis [32,33].

The mitochondria-dependent pathways involve pro-apoptotic cascades that culminate in cytochrome *c *release. CHOP (C/EBP homology protein) is one of the proteins involved, which heterodimerizes with several C/EBP family members to regulate their transcriptional activity [34]. CHOP is downstream of phosphorylation cascade of PERK and eIF-2α. CHOP has a role in the induction of cell death by promoting protein synthesis and oxidation in the stressed ER. It modulates the Bcl-2 family of proteins, GADD34 (growth arrest and DNA damage inducible protein 34), and TRB3 (tribbles-related protein 3), among other downstream proteins. After transcriptional activation by ATF4, CHOP directly activates GADD34, which promotes ER client protein biosynthesis by dephosphorylating phospho-Ser 51 of the α subunit of eIF-2α in stressed cells [35,36]. Additionally, it has been suggested that CHOP upregulates pro-apoptotic members of the BCL2 family (BAK/BAD) and downregulates the anti-apoptotic members (BCL2), causing subsequent damage to the mitochondrial membrane and releasing cytochrome *c *into the cytosol. The released cytochrome *c *in turn activates cytosolic apoptotic protease activating factor1 (APAF1), which then activates the downstream caspase-9 and caspase-3-dependent cascade [37].

A number of ER stress conditions can cause calcium release from the ER to the cytosol, Increases in cytosolic calcium can also cause activation of calpain, which induces cleavage of procaspase-12 [38]. Once activated, the catalytic subunits of caspase-12 are released into the cytosol, where they activate the caspase-9 cascade in a cytochrome *c *independent manner [39].

It has also been suggested that activated IRE1a can recruit tumor-necrosis factor receptor associated factor 2 (TRAF2), which activates procaspase-4 as a mitochondria-independent apoptotic response. Both pathways ultimately lead to the activation of the caspase cascade mediated through caspase-9 and caspase-3, resulting in cell death [40].

## 2. The unfolded protein response and its effect on tumorigenesis

A broad range of cancer-types rely on ER protein folding machinery to correctly fold key signaling pathway proteins [41]. ER stress and the UPR are highly induced in various tumors. Accumulating evidence has demonstrated that the UPR is an important mechanism required for cancer cells to maintain malignancy and therapy resistance. Identifying the UPR components that are activated or suppressed in malignancy and exploring cancer therapeutic potentials by targeting the UPR are very active research areas [7].

The UPR pathways are activated in a great variety of tumor types, and have been demonstrated to be essential for tumor cells to survive the unfriendly tumor microenvironment. There are evidence of over-expression of XBP1s (excision of a 26 nucleotide unconventional intron from XBP-1 mRNA), activation of ATF6, phosphorylation of eIF-2α, induction of ATF4 and CHOP in a variety of cancer cells. The ER chaperones GRP78/BiP, glucose-regulated protein 94 (GRP94, also known as gp96 or HSP90b1) and GRP170 were also upregulated [42]. These studies were conducted in primary human tumor cells or cell lines, and animal models with breast tumor, hepatocellular carcinoma, gastric tumor, and esophageal adenocarcinoma [42-52]. UPR and stress response in general have also been implicated in participating in inflammation-induced oncogenesis [53].

UPR is required for tumorigenesis. Animal study demonstrated that XBP1 was required for tumor growth *in vivo*. *Xbp1*^-/- ^and *Xbp1*-knockdown cells did not form tumors in mice even though their growth rate and secretion of vascular endothelial growth factor (VEGF) in response to *in vitro *hypoxia treatment were not decreased [46]. ER stress can also induce anti-apoptotic responses. The activation of glycogen synthase kinase 3β (GSK 3β) leads to phosphorylation of p53, which increases its degradation [54], therefore protects cancer cells from p53 dependent apoptosis. In addition, NFκB is activated during ER stress to induce anti-apoptotic responses [55].

Heat shock proteins were reported to assist cancer cell adaptation to oncogenesis-associated stress either by repairing damaged proteins (protein refolding) or by degrading them. Heat shock proteins have also been implicated in the control of cell growth, and in resistance to various anticancer treatments that induce apoptosis. For example, HSP90 interacts with several key proteins in promoting prostate cancer progression, including wild-type and mutated AR, HER2, ErbB2, Src, Abl, Raf and Akt [56,57]. GRP78/BiP, expressed at high levels in a variety of tumors, confers drug resistance in both proliferating and dormant cancer cells. Genetically engineered animal model with reduced GRP78 level significantly impedes tumor growth. Three major mechanisms were proposed for GRP78 mediated cancer progression: enhancement of tumor cell proliferation, protection against apoptosis, and promotion of tumor angiogenesis [58-60].

ER stress has been implicated in different stages of tumor development. The proposed mechanism is, during early tumorigenesis and before angiogenesis occurs, that activation of the UPR induces a G1 cell cycle arrest and activation of p38, both of which promote a dormant state. If the apoptotic signals are induced by the UPR during this stage of tumor development, cancer cells with mutated elements of the apoptotic pathway may evade the alternative fate of death. ER stress also induces anti-apoptotic NF-κB and inhibits p53-dependent apoptotic signals. If the balance of early cancer development tilts against cell death, ER stress can further promote the aggressive growth of these cancer cells by enhancing their angiogenic ability. One example is the increased VEGF secretion through induction of GRP170, a BiP-like protein that acts as a chaperone for VEGF [37].

## 3. The unfolded protein response and its effect on disease prognosis

GRP78 is a marker of UPR activation. An elevated GRP78 level generally correlates with higher pathologic grade, recurrence rate, and poor survival in patients with breast, liver, prostate, colon, and gastric cancers; though there are conflicting reports on lung cancer. Neuroblastoma is an apparent exception with correlation of GRP78 abundance with earlier stage and better prognosis [59,61-64].

A retrospective cohort study of 127 stage II and III breast cancer patients who were treated with Adriamycin-based chemotherapy, showed association between GRP78 positivity and shorter time to tumor recurrence [59]. Another breast cancer study showed that the UPR is activated in the majority of breast cancers and confers resistance to chemotherapy and endocrine therapy. Estrogen is known to stimulate UPR *in vitro*. UPR activation interacts with estrogen response elements and may regulate tumor growth [65].

Overexpression of GRP94 and GRP78 has been observed more often in patients with poorly differentiated lung cancer than in well or moderately differentiated tumors [66]. According to a study on adenocarcinoma of the esophagus, GRP78 and GRP94 mRNA were elevated in all tumors. Increased expression of GRP78 may be responsible for controlling local tumor growth in early tumor stages, while high expression of GRP78 and GRP94 in advanced stages was believed to be dependent on other cellular stress reactions such as glucose deprivation, hypoxia, or the hosts' immune response [67]. Up-regulated expression of GRP78 and GRP94 was also reported in gastric carcinoma, which was associated with aggressive tumor growth and poor prognosis [68].

Heterozygous GRP78 mice with half of wild-type GRP78 level are comparable to WT siblings in tumor growth and development. The tumor progression was significantly impeded in these mice as exemplified by a longer latency period, reduced tumor size, and increased tumor apoptosis. Reduction of GRP78 in cancer xenograft animal model inhibited tumor formation and growth [69].

XBP1s is a *trans*-activator of UPR signaling. High XBP1s level is associated with increased tumor growth, resistance to anti-estrogen therapy and poor patient survival [70,71]. In a B cell-specific XBP1s-overexpressing transgenic mouse model, multiple myeloma developed spontaneously, highlighting the importance of UPR in tumorigenesis [72].

## 4. Therapeutic targeting of unfolded protein response in cancer

The accumulation of unfolded proteins triggers the UPR, which mediates the inhibition of general protein synthesis but increases expression of several transcription factors that activate genes encoding ER stress-inducible molecular chaperones, transcription factors and signal pathway proteins. Most normal cells are not undergoing active "stress" response, and the UPR pathways remain in a quiescent state in these cells. This discrepancy between tumor cells and normal cells offers an advantage for the agents that target the UPR to achieve the specificity in cancer therapy. The therapeutic potential of targeting the UPR components in cancer mainly involves two approaches: induction of accumulation of misfolded protein in ER to overload the unfolded protein response, and inhibition of UPR adaptive and antiapoptotic pathways to prevent cells from adapting to stressful conditions leading to cell death. In the following paragraphs, we will discuss some examples of agents that are being developed as cancer therapeutics (Table 1).

**Table 1:** Examples of UPR-targeted cancer drugs in development

Drug	Classification/Mechanism	Development Stage	Disease Indication	Reference
Bortezomib	Proteasome inhibitor	FDA approved	Multiple myeloma, mantle-cell lymphoma	San *et al. *[97]

NPI-0052 (salinosporamide A)	Irreversible proteasome inhibitor	Phase I clinical trials	Multiple Myeloma, Advanced malignancies	Chauhan *et al. *[98]

Carfilzomib (PR-171)	Selective proteasome inhibitor	Phase I, II, III clinical trials	Multiple Myeloma, Waldenstrom's Macroglobulinemia	O'Connor *et al. *[99] Lee *et al. *[100]

PS-341	Selective proteasome inhibitor	Phase II	Multiple Myeloma	Richardson *et al. *[101]

CEP-18770	Proteasome inhibitor	Phase I, II clinical trials and preclinical studies	multiple myeloma, Non- Hodgkin's lymphoma	Piva *et al. *[102]

Tanespimycin (17-AAG, (17-Allylamino-17-demethoxygeldanamycin), KOS-953)	HSP90 Inhibitor	Phase I, II, III clinical trials	Gastrointestinal stromal tumors, breast cancer, gynecological, leukemia, lymphoma, melanoma, prostate, renal, thyroid carcinoma, melanoma	Richardson *et al. *[103,104] Heath *et al. *[105] Pacey *et al. *[106]

Alvespimycin (KOS-1022, 17-DMAG)	HSP90 Inhibitor	Phase I clinical trials and preclinical studies	Acute myeloid leukemia, advanced carcinoma	Kummar *et al. *[107] Lancet *et al. *[108] Pamanathan *et al. *[109] Zismanov *et al. *[110]

Retaspimycin (IPI-504)	HSP90 Inhibitor	Phase II clinical trials	Gastrointestinal stromal tumors, nonsmall cell lung, prostate	Hanson *et al. *[111]

PU-H71	HSP90 Inhibitor	Preclinical studies	Breast cancer, myeloma, myeloproliferative disorder	Usmani *et al. *[84] Caldas-Lopes *et al. *[112] Marubayashi *et al. *[113]

SNX-2112	HSP-90 inhibitor	Preclinical studies	Gastric cancer	Bachleitner-Hofmann, *et al. *[114]

Eeyarestatin I (EerI)	Inhibitor of ER-associated degradation (ERAD)	Preclinical studies		Cross *et al. *[115]

Versipelostatin	GRP78 inhibitor	Preclinical studies		Matsuo *et al. *[87]

(-)-epigallocatechin gallate (EGCG)	GRP78 inhibitor	Preclinical studies	Breast carcinoma	Luo *et al. *[116]

Epidermal growth factor (EGF)-SubA	GRP78-targeting cytotoxin	Preclinical murine animal models	Prostate tumor	Backer *et al. *[90]

Irestatins	IRE1α inhibitor	Preclinical studies	Multiple Myeloma,	Feldman *et al. *[117]

Delta(9)-Tetrahydrocannabinol (THC)	Cannabinoid, activates ER stress and autophagy	Phase I clinical trial	Glioblastoma multiforme	Guzmán *et al. *[118]

### 4.1. Targeting induction of unfolded protein response

#### Proteasomal inhibitor

Proteasomal degradation of misfolded proteins retrotranslocated from the ER to the cytosol represents the final step in ERAD. Bortezomib (Velcade, PS-341), a boronic acid derivative, was the first proteosome inhibitor to be developed successfully for anti-cancer therapy. Although the drug probably has multiple mechanisms of action, proteasomal inhibition causes an additional burden of unfolded proteins in the ER. This explains the high efficacy of bortezomib treatment against types of cancer cells in which the ER is already predisposed with a considerable protein load. In multiple myeloma cell lines, Bortezomib rapidly induced components of the proapoptotic UPR, including PERK, the ER stress-specific eIF-2α kinase, ATF4 and its proapoptotic target, CHOP. The amount of immunoglobulin subunits retained within multiple myeloma cells correlated with their sensitivity to proteasomal inhibitors [73].

Bortezomib treatment has a cytotoxic effect on various other cancer types such as breast, colorectal, ovarian, pancreatic, prostate, lung and oral cancer. It has been approved by the FDA for the treatment of relapsed multiple myeloma, and recently for relapsed mantle cell lymphoma. Combination chemotherapy regimens with Bortezomib have been developed, leading to unprecedented high remission rates in the frontline treatment or in the relapsed setting for multiple myeloma. The combination of proteasome inhibition with novel targeted therapies is an emerging field in oncology [74].

#### ERAD inhibitors

As a part of ER quality control mechanism, misfolded or unassembled proteins are retained in the ER and subsequently degraded by ERAD. In the ERAD pathway, molecular chaperones and lectin-like proteins are involved in the identification of misfolded proteins. ER-resident reductases cleave disulfide bonds in these proteins to facilitate retrograde transport to the cytosol. Furthermore, the AAA(+) adenosine triphosphatase withdraws them from the retrotranslocation channel to the cytosol where they are degraded by the ubiquitin/proteasome system [75].

Defects in ERAD cause the accumulation of misfolded proteins in the ER and thus trigger ER stress and UPR. Eeyarestatin I (EerI), a chemical inhibitor that can block ERAD, has been shown to have preferential cytotoxic activity against cancer cells. EerI targets p97 (a cytosolic ATPase involved in polyubiquitinated proteins transportation) complex to inhibit deubiquitination of p97-associated ERAD substrates, which is required for the degradation process [76].

#### PDI inhibitors

Protein disulfide isomerase (PDI) is one of the most abundant ER proteins and maintains a sentinel function in organizing accurate protein folding. PDIs are key protein folding catalysts activated during UPR [77]. Treatment of cells with O(2)-[2,4-dinitro-5-(N-methyl-N-4-carboxyphenylamino)phenyl]1-(N,N- methylamino)diazen-1-ium-1,2-diolate (PABA/NO) resulted in a dose-dependent increase in intracellular nitric oxide that caused S-glutathionylation and therefore inhibition of PDI. PABA/NO activates the UPR and causes translational attenuation, phosphorylation and activation of PERK, and its downstream effector eIF2α in human leukemia (HL60) and ovarian cancer cells (SKOV3). There was also evidence for *Xbp1 *mRNA splicing and transcriptional activation of the ER resident chaperones GRP78 and GRP94. Stimulating UPR may be linked with the cytotoxic potential of PABA/NO in cancer cells [78].

### 4.2. Targeting ER chaperones/heat shock proteins

#### HSP90 inhibitor

Under conditions of cellular stress, cells upregulate chaperones to prevent protein misfolding and degradation. All three ER-membrane bound sensors are heavily reliant on the protein chaperone functions of the HSP90 complex. The interaction between the heat shock protein family and the key proteins in the UPR pathway may, in part, be mediated by their destabilizing effect on UPR proteins and increased accumulation of misfolded proteins.

Myeloma cell study demonstrated that HSP90 inhibitors, 17AAG (17-allylamino-17-demethoxygeldanamycin) and radicicol, similar to tunicamycin (TM) and thapsigargin (TG) (known UPR activators), are capable of activating all three branches of the UPR. All drugs inhibited proliferation and increased expression levels of the molecular chaperones BiP and GRP94. Unlike TG and TM, the HSP90 inhibitors activate a caspase-dependent cell death pathway [79]. 17AAG can induce the formation of 'intracellular inclusions' in breast cancer cells. In myeloma cells, these inclusions are comprised of aggregations of misfolded immunoglobulin light chains and analysis of protein samples taken from 17AAG-treated cells suggest that exposure to HSP90 inhibitors alters the expression of LC3 (microtubule-associated protein 1 light chain 3, a reliable marker for autophagosome formation), consistent with autophagosome formation [80-82].

Study demonstrated analogous effects of HSP90 inhibitor, 17AAG in the colon cancer cell line HCT116 indicating that they utilize the UPR in a similar manner to multiple myeloma [41]. A recent phase II trial was done using the HSP90 inhibitor, 17-AAG in fifteen melanoma patients with measurable disease. 17-AAG was administered i.v. once weekly for 6 weeks at 450 mg/m^2^. No objective responses were observed. Western blot analysis of tumor biopsies showed an increase in HSP70 and a decrease in cyclin D1 expression in the posttreatment biopsies. UPR components were not analyzed in this study. More potent HSP90 inhibitor or a formulation that are soluble and can be administered chronically for a more prolonged suppression effect on UPR may be necessary to be clinically beneficial [83]. A phase III clinical trial is ongoing to evaluate the utility of 17-AAG in multiple myeloma patients. There are also Phase II clinical trails in breast cancer and non-small cell lung carcinoma. PU-H71, a novel purine scaffold HSP90 inhibitor, has shown interesting preclinical activity against myeloma [84].

#### Grp78/BiP inhibitor

Levels of Grp78/BiP are commonly raised in solid tumors and cancer cell lines [85]. Versipelostatin (VST) and analogues, novel macrocyclic compound and GRP78/BiP inhibitor, showed promise in solid tumors [86]. VST has demonstrated selective cytotoxicity to glucose-deprived tumor cells by preventing the unfolded protein response. It was shown to inhibit GRP78 induction and the expression of the UPR transactivators XBP1 and ATF4. Eukaryotic initiation factor 4E-binding protein 1 (4E-BP1), a negative regulator of eukaryotic initiation factor 4E-mediated protein translation, plays a role in the UPR-inhibitory action of VST. Aberrant activation of 4E-BP1 prevents induction of the GRP78 and ATF4 [7,87-89].

Treatment of glioma cells with another GRP78 inhibitor, epigallocatechin gallate (EGCG,) which targets the ATP-binding domain of GRP78 and blocks its UPR protective function, sensitizes glioma cells to chemotherapy agent temozolomide [85]. Additionally, an engineered fusion protein, epidermal growth factor-SubA (EGF-SubA), a chaperone-targeting cytotoxin, was reported to be highly toxic to growing and confluent epidermal growth factor receptor-expressing cancer cells, and its cytotoxicity is thought to be mediated by rapid cleavage of GRP78 [90].

### 4.3. Inhibiting IRE1α/XBP1 pathway

#### Inhibitors of the IRE1α/XBP1 pathway

Irestatin, an inhibitor of IRE1 and the unfolded protein response, mediates inhibition of XBP1s transcription activity. The inhibition of the IRE1 endonuclease impairs the growth of malignant myeloma cells and inhibits the survival of oxygen-starved tumor cells *in vitro *and subcutaneous HT1080 tumor xenografts [91].

Trierixin, a new member of the triene-ansamycin group, isolated from the fermentation broth of *Streptomyces sp. *AC654, was shown to be a novel inhibitor of ER-stress induced cleavage of XBP1 [92]. Future work needs to be done to evaluate its activity in cancer therapy.

### 4.4. Other agents affecting unfolded protein response

IPI-504, a soluble HSP90 inhibitor, can block the unfolded protein response in multiple myeloma (MM) cells. Partial UPR is constitutively activated in plasma cell-derived MM cells. IPI-504 can potently inhibit this pathway. IPI-504 achieves this by inactivating the transcription factors XBP1 and ATF6. In addition, IPI-504 also blocks the tunicamycin-induced phosphorylation of eIF2α by PERK. The inhibitory effect of IPI-504 on the UPR parallels its cytotoxic and pro-apoptotic effects on multiple myeloma cells [93].

As discussed above, autophagy is a cellular process in which cytoplasmic materials are sequestered into autophagosomes and delivered to lysosomes for degradation or recycling. It can switch from cytoprotective role to a form of programmed cell death with persistent ER stress. Tetrahydrocannabinol (THC), the main active component of marijuana, induces human glioma cell death through stimulation of autophagy. THC induced autophagy is associated with an increased phosphorylation of eIF2α [94].

Resveratrol (RES), a natural plant polyphenol, is an effective inducer of cell cycle arrest and apoptosis in a variety of carcinoma cell types. In addition, RES has been reported to inhibit tumorigenesis in several animal models. RES causes cell cycle arrest and proliferation inhibition via induction of UPR in human leukemia K562 cell line [95].

The phytoestrogen zearalenone (ZEA), one of the most active naturally occurring estrogenic compounds in food and beverages, has also been shown recently to induce human leukemic cell apoptosis via endoplasmic stress and mitochondrial pathway [96].

## 5. Perspectives

We have highlighted the importance of UPR in tumorigenesis and provided an overview on the potential strategy in perturbing UPR in cancer treatment. URP promotes the ability of cancer cells to adapt to and survive the hostile microenvironment through activation of stress-response pathways and upregulation of chaperones. Targeting URP pathway represents a novel targeted anti-cancer approach with initial successes in clinical studies. Further understanding of the pathway should provide additional therapeutic opportunities.

Clearly, UPR and the associated molecular components are emerging as important potential targets for drugs that may be used in the treatment of cancer in which protein-folding and protein quality control play a key role in disease pathology. This area looks set to be a very exciting one in years to come. It is worthwhile to point out that protein quality control is fundamentally important for life. Thus targeted therapy towards UPR or other arms of protein quality control is by no means cancer-specific and toxicity-free. Of particular importance is the lack of understanding of the fundamental roles and mechanisms of protein quality control in development, organ function, the evolution and fitness of organism. Thus, as more pharmacological agents are being developed clinically, attention needs to be paid to the understanding of the basic mechanism of the regulation of unfolded protein response and to the discovery of important new players in the protein quality control for disease target.

## Competing interests

The authors declare that they have no competing interests.

## Authors' contributions

All authors participated in the writing of this manuscript and have approved its publication.
